# A Photonic 1 × 4 Power Splitter Based on Multimode Interference in Silicon–Gallium-Nitride Slot Waveguide Structures

**DOI:** 10.3390/ma9070516

**Published:** 2016-06-25

**Authors:** Dror Malka, Yossef Danan, Yehonatan Ramon, Zeev Zalevsky

**Affiliations:** 1 Faculty of Engineering, Department of Electro-Optics, Bar-Ilan University, Ramat-Gan 52900, Israel; yosidanan@gmail.com (Y.D.); yonathan.ram@gmail.com (Y.R.); zalevsz@eng.biu.ac.il (Z.Z.); 2 Faculty of Engineering Holon Institute of Technology (HIT), Holon 5810201, Israel

**Keywords:** optical splitter, slot waveguide, multimode interference (MMI)

## Abstract

In this paper, a design for a 1 × 4 optical power splitter based on the multimode interference (MMI) coupler in a silicon (Si)–gallium nitride (GaN) slot waveguide structure is presented—to our knowledge, for the first time. Si and GaN were found as suitable materials for the slot waveguide structure. Numerical optimizations were carried out on the device parameters using the full vectorial-beam propagation method (FV-BPM). Simulation results show that the proposed device can be useful to divide optical signal energy uniformly in the C-band range (1530–1565 nm) into four output ports with low insertion losses (0.07 dB).

## 1. Introduction

Optical power splitters play a crucial role in optical communication systems [1]. These components are significant for bringing the optical fiber to end-users [2].

Multimode interference (MMI)-based devices are important building blocks for photonic integrated circuits due to their simple structure, low excess loss, large optical bandwidth, and low polarization dependence [3,4]. The operation of photonic MMI devices is based on the self-imaging principle [3]. In a multimode waveguide, an input field profile is reproduced in single and multiple images at periodic intervals along the propagation axis of the waveguide, which practically produces self-images at different locations [5].

MMI waveguide photonic devices were used for numerous applications such as filters [6], temperature sensors [7], splitters [8,9], and couplers [10].

Slot waveguide structures are based on a combination of low-index material and high-index material [11]. The low-index layer (slot area) is surrounded by two high-index layers that enable the total internal reflection (TIR) effect in order to guide the light into the slot waveguide structure. There are no confinement losses in slot waveguide structures due the strong high power confinement inside the slot area (low-index). Therefore, there is a significant interest in designing a photonic device based on a slot waveguide structure that integrates semiconductor materials. Several studies [12,13,14,15,16,17,18] have been conducted to show the great potential of using a slot waveguide structure to design a photonic device. In addition, the fabrication of slot waveguide structures can be done with CMOS technologies for realizing silicon (Si) photonic chips [19].

Gallium nitride (GaN) has been widely used for integrating optically active nanoscale components with non-photonic devices [20]. For example, GaN components can be grown on epitaxial substrates or can be grown directly on silicon substrates [21]. GaN is well-known for its superior electrical properties, its resistance to temperature, and its potential to cover a wide spectral range.

The benefits of using GaN based on conventional waveguides to design optical splitters [22,23] and couplers [24,25] have been demonstrated. Recently, researchers have demonstrated the potential of using GaN based on a slot waveguide for transmitting light in the visible range (400–800 nm) with little transmission loss (0.4–1.0 dB/cm) [26]. A MMI-device-based slot waveguide are very sensitive to the variation of the effective refractive index, which can influence the performance. Therefore, it is better to use a Si–GaN slot waveguide that has a low index difference compared with other materials (e.g., Si-alumina [15] and Si-silica (SiO_2_) [27]) that were found to be suitable for a MMI-device-based slot waveguide. Thus, it is clear that using the GaN as the slot material can lead to better performances.

In this paper, we introduce a unique design of a 1 × 4 power splitter based on an MMI coupler in a Si–GaN slot waveguide structure. Tapered waveguides were integrated into the input/output of the MMI coupler to reduce the excess loss. Numerical investigations were carried out on the geometrical parameters of the device in order to obtain a self-imaging effect, strong power confinements inside the slot area, and uniform splitting of the optical signal at the output ports. The simulations were done using the full vectorial-beam propagation method (FV-BPM) [19].

The device operates at a wavelength of around 1550 nm with an 81-nm full width at half maximum (FWHM). Therefore, this device can be used in an optical networking system to split the energy in the C-band range.

## 2. The 1 × 4 Power Splitter Structure and the Theoretical Aspect

**Figure 1:**
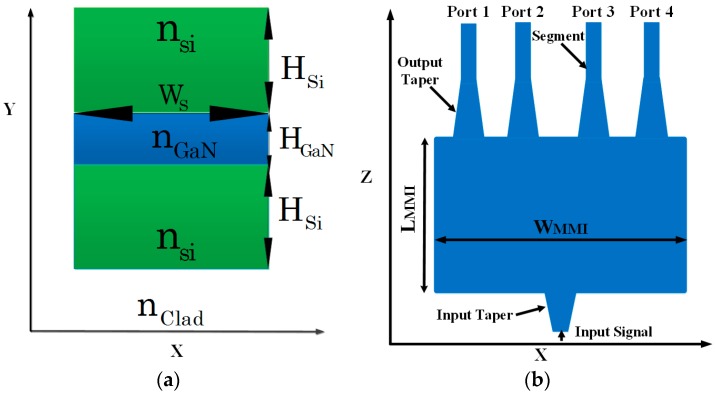
Schematic illustration of the optical 1 × 4 power splitter: (**a**) *x*–*y* plane; (**b**) *x*–*z* plane.

Figure 1a shows a schematic sketch of our proposed 1 × 4 power splitter at the *x*–*y* plane. In Figure 1a, the green areas represent pure Si, blue area represents GaN, and the white area represents pure SiO_2_. The refraction index values for the operated wavelength (1550 nm) are n_si_ = 3.48 (Si), n_GaN_ = 2.305 (GaN), and n_clad_ = 1.444 (SiO_2_).

**Figure 2:**
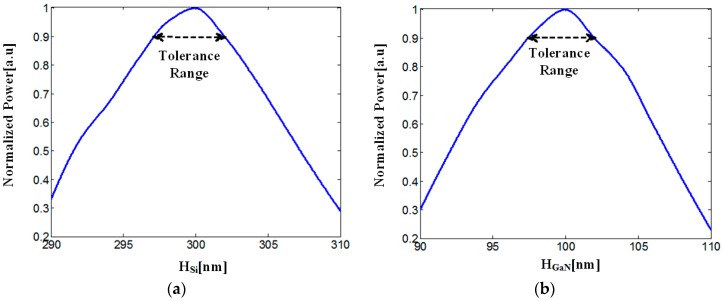
Normalized power at the slot area as a function of the geometrical parameters: (**a**) H_Si_; (**b**) H_GaN_.

**Figure 3:**
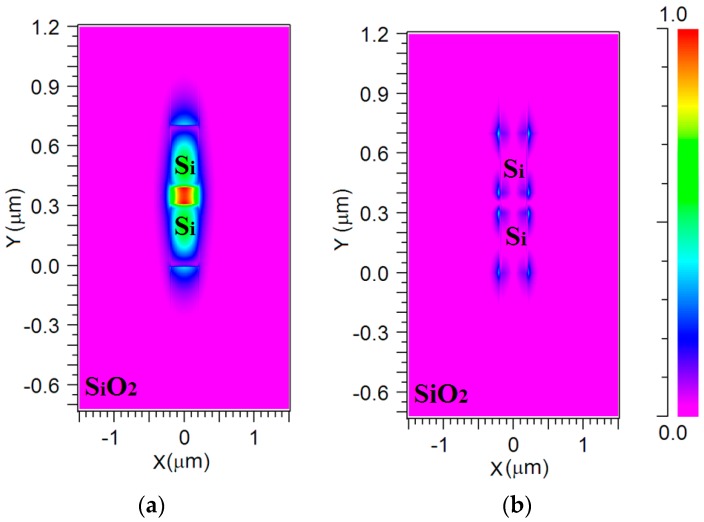
Quasi-transverse magnetic (TM) fundamental field profile mode for the 1 × 4 power splitter: (**a**) Major mode E_y_; (**b**) minor mode E_x_.

The optimal geometric values (see Figure 2a,b) that have been found suitable to the Si–GaN slot waveguide structure are H_Si_ = 300 nm (height of Si layer), H_GaN_ = 100 nm (height of GaN layer), and W_S_ = 400 nm (width of layer Si/GaN). These values have been chosen in order to enable a strong confinement of the electric field inside the slot area (see Figure 3).

Figure 1b shows a cross-sectional view of the *x*–*z* plane. The 1 × 4 power splitter is based on one input taper, four output tapers, one MMI coupler, and four waveguide segments. The length of the input/output taper is 2 µm/5 µm, and the width of the input/output taper varies from 0.4–0.6 µm/0.6–0.4 µm, respectively.

The width of the segment waveguide is 0.4 µm with a length of 5 µm. The gap width between the two ports at the output is 0.7 µm.

The operation principle of the 1 × 4 MMI power splitter in the Si–GaN slot waveguide structure is based on the self-imaging effect [3] and the TIR effect. We will denote L_π_ by the beat length of the first two modes propagating through the MMI. This length can be approximated with [3]

(1) $$L_{\pi }\approx \frac{4n_{eff}(\lambda )W_{e}^{2}}{3\lambda },$$

where n_eff_ is the effective refractive index of the core in the slot waveguide (GaN and Si), which is solved by the FV-BPM. W_e_ is the effective width of the MMI coupler, and λ is the operating wavelength. The W_e_ for the case of transverse magnetic (TM) mode is given by [3]

(2) $$W_{e}=W_{MMI}+\frac{\lambda }{\pi }{\left(\frac{n_{clad}}{n_{eff}}\right)}^{2}\frac{1}{\sqrt{\left(n_{eff}^{2}-n_{clad}^{2}\right)}},$$

where the width of the MMI coupler is W_MMI_, and its size is 5 µm. This size has been chosen in order to reduce the L_π_ value. Secondly, this size extremely depends on the number of MMI ports at the output.

The length of the MMI coupler is given by [3]

(3) $$L_{MMI}=\frac{3pL_{\pi }^{\lambda }}{4N},$$

where p is a positive integer, and N is the number of ports at the output of the MMI coupler. In our case, p = 1 and N = 4.

The value of L_MMI_ can be further optimized by choosing certain geometrical parameters of the MMI waveguide and by optimizing the operation wavelength.

## 3. Simulation Results

The simulations of the 1 × 4 power splitter structure were performed using the FV-BPM based on the RSoft Photonics CAD Suite software (San Jose, CA, USA). Figure 3a,b show the profile mode of the electric fields E_y_ and E_x_ for a wavelength of 1550 nm.

Figure 3a shows a strong power confinement (red color) inside the slot area. Therefore, the light can be guided in the proposed design without confinement losses.

The normalized power in the slot area is shown in Figure 3a,b as a function of the geometrical parameters H_Si_ and H_GaN_. The optimal tolerance values of the parameters H_Si_ and H_GaN_ were set between 90% and 100% of the normalized power (black dashed line).

Figure 3a,b show that the tolerance values of H_Si_ and H_GaN_ are around 5/6 nm and 4/5 nm, respectively.

By solving the major mode E_y_, we have found the value of n_eff_ that is suitable for the operated wavelength, and its value is 2.89. This value is used to calculate the L_π_ and the W_e_. Solving Equations (1) and (2) has shown that the values are 64.16 µm and 5.08 µm, respectively.

**Figure 4:**
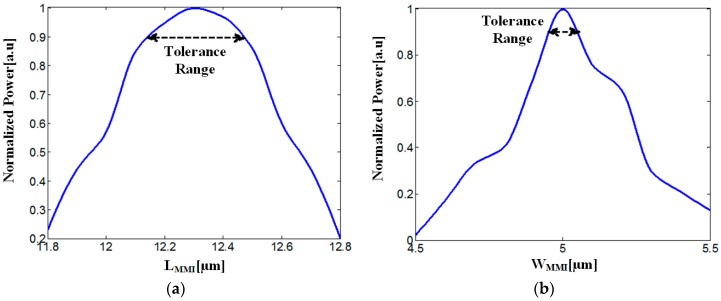
Normalized power as function of the geometrical multimode interference (MMI) coupler parameters: (**a**) L_MMI_; (**b**) W_MMI_.

Solving Equation (3) has shown that the value of L_MMI_ is 12.03 µm. This value has been optimized by applying FV-BPM simulations. The optimization of the L_MMI_ and W_MMI_ is shown in Figure 4a,b. It can be noticed from Figure 4a,b that the optimal values of the L_MMI_ and W_MMI_ are 12.3 µm and 5 µm, respectively. These values lead to the best performance of the designed structure. The optimal tolerance values of the parameters L_MMI_ and W_MMI_ were set as in Figure 2.

Figure 4a,b show that the tolerance values of L_MMI_ and W_MMI_ are around 0.3/0.4 μm and 65/85 nm, respectively.

**Figure 5:**
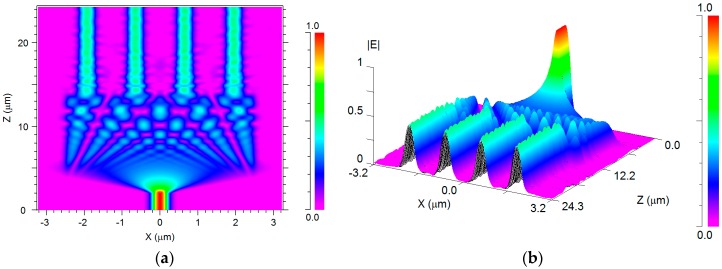
The propagation profile of the electric field for the 1 × 4 MMI power splitter at *x*–*z* plane: (**a**) 2D; (**b**) 3D.

The absolute value of the propagating electric field inside the 1 × 4 MMI power splitter at *x*–*z* plane is shown in Figure 5a,b. Figure 5a shows that indeed the intensity of the optical signal is equally split at z = 15 µm into four beams guided to the output ports of the MMI coupler. For more clarity, this result is also represented in three dimensions in Figure 5b.

**Figure 6:**
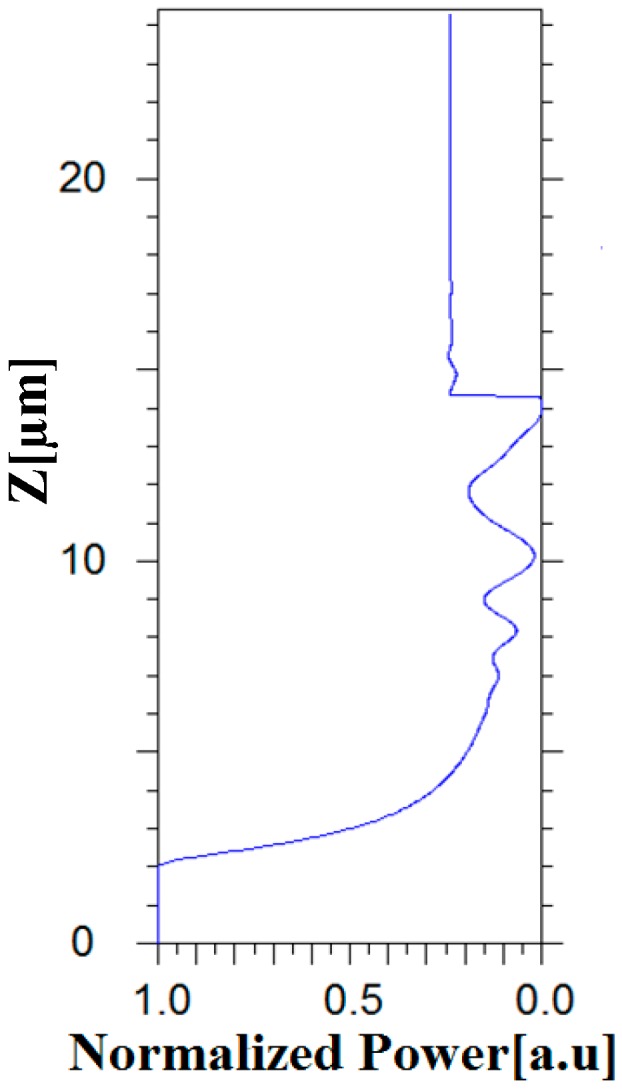
Propagation distance *Z* as a function of power propagation.

In addition, the normalized power of the optical signal (1550 nm) along the propagation axis (*z*-axis) was analyzed via FV-BPM simulations. Figure 6 shows the distribution of the normalized power value along the propagation of the optical signal in the proposed design. It can be noticed from Figure 6 that the power value in each port is exactly 24.6% of the total power.

To examine the performance of the proposed 1 × 4 power splitter, we have calculated the insertion losses, which are given by

(4) $$Losses[dB]=-10Log_{10}\left(\frac{Np_{out}}{P_{in}}\right),$$

where P_in_ is the power in the input waveguide taper, and P_out_ is the power in the output port. The insertion losses for all four ports are 0.07 dB. Therefore, the transfer energy is almost 100% from the input waveguide taper into all the four output ports. Thus, the proposed 1 × 4 power splitter is energetically efficient.

**Figure 7:**
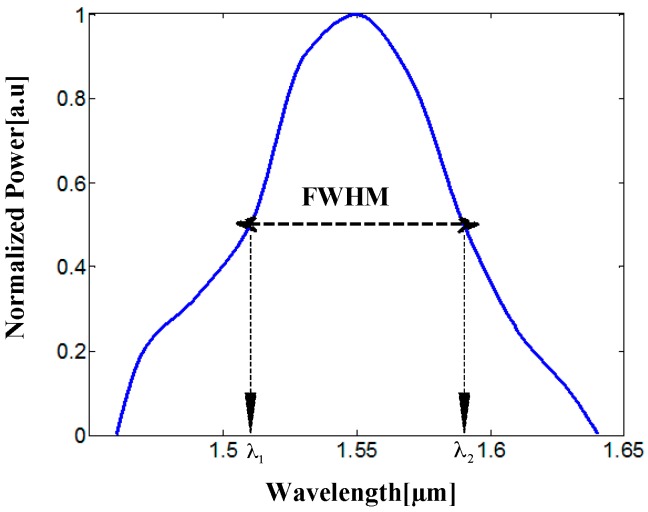
Normalized power as function of wavelength.

In addition, a Matlab code combined with FV-BPM simulations was performed to determine the power splitter properties of the designed structure. Figure 7 shows the spectral transmission results for different wavelengths close to the wavelength of the optical signal (1550 nm).

The FWHM of the power spectrum is given by

(5) $$FWHM=\lambda_{2}(P=0.5)-\lambda_{1}(P=0.5)$$

where P is the normalized power. Figure 7 shows that the FWHM for each port is around 81 nm (1510–1591 nm). It can be seen in Figure 7 that the FWHM of the power splitter is suitable for the C-band (1530–1565 nm) range of the optics communication field.

## 4. Preliminary Fabrication Results

**Figure 8:**
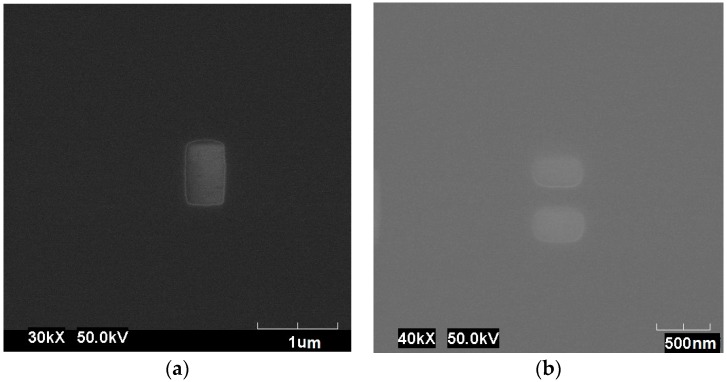
Preliminary fabrication of the silicon–gallium nitride (Si–GaN) slot waveguide: (**a**) The first layer of GaN over the Si–silica (SiO_2_) sample. (Dim.: 400 × 700 nm); (**b**) the second step of exposure for Si.

We first spin-coated a sensitive E-beam resist (poly-methyl-methaclyrate A4) layer and baked it for 120 s on a hotplate at 180 °C. The desired pattern was created by exposing the resist layer to a high-resolution E-beam (CRESTEC CABLE-9000C lithography system) (Shizuoka, Japan). The exposure level was done with a fine alignment using an accurate marking technique. Then, a thermal evaporation system (Nano 36 vacuum by K.J. Lesker) was used to deposit 50 nm of GaN, as shown in Figure 8a. The sample was immersed in acetone for 3 h in order to remain with the wanted pattern (this technique known in the literature as “lift-off”). The sample was masked again by maintaining the previous alignment step as shown in Figure 8b. Finally, we deposited another 50 nm of intrinsic Si and repeated the “lift-off” etching in order to attain the final structure of the slot waveguide.

## 5. Conclusions

To conclude, we have shown that a 1 × 4 power splitter can be implemented on MMI in a Si–GaN slot waveguide structure. Through simulation results, it was shown here that the energy of the optical signal at a wavelength of 1550 nm can be split after a propagation length of about 15 µm, with equal intensity in each output port.

The FWHM is 81 nm for each port, and the insertion losses of the proposed device are below 0.07 dB for all four ports. Therefore, this splitter can be used in an optical networking system that works on the entire C-band range.

Although only the splitter configuration is considered in this work, the proposed device can also operate as a combiner (coupler) by reversing the direction of the light wave propagation.

This design has the potential to integrate with techniques of CMOS technology for realizing a photonic chip due to the use of semiconductor materials (i.e., Si–GaN).
